# A Novel Highly Efficient Device for Growing Micro-Aerophilic Microorganisms

**DOI:** 10.3389/fmicb.2019.00534

**Published:** 2019-03-19

**Authors:** Maxime Fuduche, Sylvain Davidson, Céline Boileau, Long-Fei Wu, Yannick Combet-Blanc

**Affiliations:** ^1^Aix Marseille University, IRD, CNRS, Université de Toulon, Marseille, France; ^2^Aix Marseille University, CNRS, LCB, Marseille, France

**Keywords:** microaerobes, MOCD, magnetotactic bacteria, *Magnetospirillum gryphiswaldense* MSR-1, *Magnetospira* sp. QH- 2

## Abstract

This work describes a novel, simple and cost-effective culture system, named the Micro-Oxygenated Culture Device (MOCD), designed to grow microorganisms under particularly challenging oxygenation conditions. Two microaerophilic magnetotactic bacteria, a freshwater *Magnetospirillum gryphiswaldense* strain MSR-1 and a marine *Magnetospira* sp. strain QH-2, were used as biological models to prove the efficiency of the MOCD and to evaluate its specifications. Using the MOCD, growth rates of MSR-1 and QH-2 increased by four and twofold, respectively, when compared to traditional growing techniques using simple bottles. Oxystat-bioreactors have been typically used and specifically designed to control low dissolved oxygen concentrations, however, the MOCD, which is far less sophisticated was proven to be as efficient for both MSR-1 and QH-2 cultures with regard to growth rate, and even better for MSR-1 when looking at cell yield (70% increase). The MOCD enables a wide range of oxygenation conditions to be studied, including different O_2_-gradients. This makes it an innovative and ingenious culture device that opens up new parameters for growing microaerobic microorganisms.

## Introduction

There are many microorganism-culture methods dedicated to aerobes and anaerobes (Hungate, 1969; Macy et al., 1972), but a simple, low cost, and effective culture facility for routinely growing microaerobic microorganisms (also called microaerobes) is still lacking. As a result, despite the ubiquity of these strains and the wide variety of natural habitats in which they are found, characterized by low oxygen tensions and varied oxygen gradients, little is known about microaerobes.

There are many habitats where O_2_-gradients are systematically present and play a major role in microbial ecology. Amongst these are the microoxic zones found in animals and plants which host microaerophilic bacteria (Brune et al., 2000). For example, sharp O_2_ gradients have been observed in root nodules of *Leguminosae* where bacterial nitrogen fixation takes place (Minchin et al., 1985; Kuzma et al., 1993; Soupène et al., 1995), and O_2_-gradients have been found between the epithelium walls and lumens in the gastro-intestinal tracts of animals such as snails (Charrier and Brune, 2003), termites (Brune et al., 1995; Wertz and Breznak, 2007), and mammals (Wiles et al., 2006; Marteyn et al., 2010; Van den Abbeele et al., 2011). Moreover, O_2_-gradients have also been described in soil aggregates (Tiedje et al., 1984), marine snow (Alldredge and Cohen, 1987; Ploug, 2001; Ploug et al., 2008), biofilms and sediments (Kühl et al., 2007; Fenchel and Finlay, 2008; Stewart and Franklin, 2008). It is also important to consider the oxygen minimum zones (OMZs) in the water columns within the oceans, which are now regarded to be important bellwethers for environmental changes in response to human activities (Falkowski et al., 2011).

Until recently, the generic term “microaerophile” has been used to describe microorganisms requiring atmospheres low in O_2_ (less than 21%) for optimal growth (Krieg and Hoffman, 1986; Bury-Moné et al., 2006). It is now recognized that this phenotypic trait greatly differs from one species to another and even from one microbiologist to another. Terms used to define microorganism phenotypes with respect to oxygen include obligate aerobes, facultative anaerobes, aerotolerant anaerobes, obligate anaerobes, and more recently nanoaerobes (Baughn and Malamy, 2004). Compared to these, the microaerophile “definition” is considered to be too restrictive and inaccurate. For instance, even though some facultative anaerobes and nanoaerobes are able to consume oxygen at very low tensions, they are rarely qualified as “microaerophile.” To clarify the situation, Morris and Schmidt recently revisited the “microaerophile” concept and proposed the term “microaerobe” to characterize any microorganism able to respire oxygen within microoxic environments by using high-affinity terminal oxydase (e.g., cytochrome oxidases *cbb3* and *ba3*) (Morris and Schmidt, 2013).

Today, few specific methods are available to routinely grow microaerobes. They consist of either semisolid culture media to develop O_2_-gradients or solid or liquid media incubated under different low-oxygen atmospheres (Krieg and Gerhardt, 1994). These techniques are suitable for carrying out a large number of laboratory experiments, but do not enable us to study the effect of oxygen supply on these strains. To meet the challenge of O_2_-supply to microaerobe cultures, a method for specific control of oxygen partial pressure (pO_2_) has been designed and implemented in laboratory bioreactors. These oxystat bioreactors enable the cultivation of a few tough microaerobes of medical [*Helicobacter pylori* (Marchini et al., 1994; Deshpande et al., 1995), and *Campylobacter jejuni* (Verhoeff-Bakkenes et al., 2008)], and biotechnological interests [*Magnetospirillum* species (Heyen and Schüler, 2003; Schübbe et al., 2006; Sun et al., 2008; Xie et al., 2009)]. Although ideal for addressing eco- or cell-physiology topics, the sophisticated oxystat culture-systems are unsuitable for routine cultures or experiments requiring various physicochemical conditions as they are expensive, time-consuming and hard to implement.

In order to meet the expectations of microbiologists and to address the major scientific challenge related to the role of microaerobes in the biosphere, the Micro-Oxygen Culture Device (MOCD) has been developed. This cheap and handy device should enable us (the scientist) to grow any type of microorganism with specific O_2_-requirements. Two magnetotactic bacteria, *Magnetospirillum gryphiswaldense* (strain MSR-1) from fresh water and *Magnetospira* sp. (strain QH-2) from sea water, were chosen as biological models to demonstrate both the effectiveness and adaptability of the MOCD to establish various specific oxygenation conditions. We selected magnetotactic bacteria because of the very distinct and specific oxygenation conditions required for cell growth on the one hand and magnetite formation on the other. Magnetite formation always requires low O_2_-concentrations [from quasi-anoxia to 20 μM dissolved oxygen for MSR-1 (Blakemore et al., 1985; Schüler and Baeuerlein, 1998; Heyen and Schüler, 2003; Sun et al., 2008)], whereas the O_2_-concentration required for cell growth is species-dependent.

MSR-1 strain, described as a facultative anaerobic (Schleifer et al., 1991; Li et al., 2012), optimally grows from 0.2 to 210 μM dissolved oxygen (Heyen and Schüler, 2003; Bazylinski and Frankel, 2004; Sun et al., 2008). QH-2 strain, described as microaerophilic (Zhu et al., 2010; Ji et al., 2014), optimally grows from 2.0 to 40 μM dissolved oxygen (unpublished results). As a reminder, 284 μM is the saturated O_2_-concentration in water under ambient conditions (O_2_-atmosphere 21% at 20°C) (Benson and Krause, 1984).

## Materials and Methods

### Determination of the O_2_-Permeability Coefficients for PharMed^®^, Versilic-Silicone^®^, and Iso-Versinic Viton^®^ Flexible Tubes

The experimental setup (Figure 2B) consisted of a reactor, containing anoxic deionized water connected to a small end-flask equipped with an O_2_-probe (Mettler Toledo InPro 6800, Switzerland). The connection was made using a flexible tube permeable to oxygen. The reactor was a 2.6-L double-jacket glass tank (Pierre Guérin, France) with a 2.0-L working volume. It was equipped with a stirrer driven by two axial impellers, and a sensor to monitor temperature (Prosensor pt 100, France). During the experiments, the anoxic condition within the water was maintained with an incoming O_2_-free N_2_ gas stream continuously injected at a constant flow rate of 100 mL min^-1^ (via a range 0–100 mL min^-^1 mass-flow meter Bronkhorst, Netherlands) through a nozzle immersed in the bioreactor. The anoxia was continuously monitored using an O_2_-probe-reactor (Mettler Toledo InPro 6800, Switzerland). The temperature of the water in the reactor was set at 25°C using water circulation in the double jacket (Julabo F25, France). To determine the O_2_-permeability of the flexible tube material, anoxic water was pumped at a constant flow rate from the reactor to the end-flask by a peristaltic pump (Gilson Minipuls, United States). Therefore, the dissolved oxygen measured by the second O_2_-probe could only come from oxygen that had diffused through the wall of the flexible tube and this was dependant on tube-material, -geometrical features, and pump flow rate. The pump flow rate was continuously checked via the change in weight of the end-flask which was measured on a balance (Sartorius CP 4201). All this equipment was connected to a Wago Programmable Logical Controller (PLC) (France) via a serial link (RS232/RS485), a 4–20 mA analog loop or a digital signal. The PLC was connected to a computer for process monitoring and data acquisition (BatchPro software, Decobecq Automatismes, France).

The O_2_- permeability of PharMed^®^, Versilic-silicone^®^, and Iso-Versinic Viton^®^ were assessed using flexible tubes made with these materials. The internal diameter (*d*), and tube-wall-thickness (*th*) were 1.6 mm × 1.6 mm, 1.0 × 1.0, and 1.0 × 1.0, respectively. Both tube-length (*L*) (range from 7 to 1000 cm) and pump flow rate (*Q*) (range from 0.9 to 7.2 cm^-3^ min^-1^) were used as variables. Unless otherwise indicated, the oxygen concentration (*C*^in^) at the tube-inlet was estimated to be zero due to the anoxic nature of the pumped water. For the experiments performed with the shorter length of Versilic-silicone^®^ tube (7 cm), a 45 cm (*L*) of Iso-Versinic Viton^®^ tube had to be connected upstream and arranged around the rotor-pump. In this case, *C*^in^-value was calculated by taking into account the water flow rate, the geometrical features, and O_2_-permeability coefficient of the Iso-Versinic Viton^®^ tube as previously determined. In the equations presented in the “results section,” the unit used for the coefficient of oxygen permeability “*P*” was cm^3^ (oxygen volume transferred at 25°C) × mm (thickness) × min^-1^ × cm^-2^ (surface) × cm^-1^ Hg (O_2_-partial pressure). The unit for the constant of Henry’s law “*Kh* = 3.97 × 10^-4^” (Benson and Krause, 1984) was cm^3^ O_2_ (gas) × cm^-3^ H_2_O × cm^-1^ Hg (O_2_-partial pressure). The units were in cm for the tube internal diameter “*d*” and the tube length “*L*,” in mm for the wall-tube thickness “*th*,” and in cm^3^ min^-1^ for the water flow rate “*Q*.”

### Routine Cultivations of MSR-1 and QH-2 Strains

The *M*. *gryphiswaldense* strain MSR-1 was obtained from the DSMZ (DSM 6361^T^) and the *Magnetospira* sp. strain QH-2 was provided to us by Dr. Xiao’s lab (Laboratory of Marine Ecology and Environmental Sciences, Institute of Oceanology, CAS, China) (Schleifer et al., 1991; Zhu et al., 2010; Ji et al., 2014).

The MSR-1 strain culture medium (Schleifer et al., 1991) contained (per liter of deionized water): MgSO_4_ 0.05 g; K_2_HPO_4_ 0.4 g; NH_4_Cl 0.16 g; NaNO_3_ 0.23 g; Yeast Extract (Fluka Biochemical, Spain) 0.2 g; and Balch trace-mineral-element solution (Balch et al., 1979) 10.0 mL. After adjusting the pH to 6.8 with 1 mol L^-1^ KOH, the medium was distributed at a rate of 50 mL per 155-mL serum bottle. The bottles were then sealed with rubber caps, sterilized by autoclaving at 120°C for 20 min, and stored at room temperature. Before inoculation, the bottles were supplemented with 0.1 mL of the Balch vitamins stock-solution (Balch et al., 1979), 1.4 mL of sodium DL-lactate stock-solution (1 mol L^-1^), and 0.1 mL of ferric quinate stock-solution (40 mmol L^-1^). They were then inoculated with 5.0 mL of recent MSR-1 culture and incubated at 28°C in a static incubator.

The QH-2 strain culture medium (Zhu et al., 2010; Ji et al., 2014) contained (per liter of deionized water): NaCl 19.5 g; CaCl_2_ 1.8 g; MgSO_4_ 1.0 g; NH_4_Cl 1.0 g; Peptone (Difco, United States) 1 g, KCl 0.6 g; Na_2_S_2_O_3_ 0.16 g; Hepes 2.4 g; and Balch trace-mineral-element solution 10.0 mL. After adjusting the pH to 7.2 with 1 mol L^-1^ KOH, the medium was distributed at a rate of 70 mL per 155-mL serum bottle. The bottles were then sealed with rubber caps, sterilized by autoclaving at 120°C for 20 min and stored at room temperature. Before inoculation, the culture medium was supplemented with 0.1 mL of Balch vitamins stock-solution, 0.8 mL of sodium succinate stock-solution (1 mol L^-1^), and 0.1 mL of ferric quinate stock-solution (20 mmol L^-1^). They were then inoculated with 5.0 mL of recent QH-2 culture and incubated at 26°C in a static incubator.

### Preparation of the MOCD-Bottles for MSR-1 and QH-2 Cultivations

#### Determination of Optimal Volume Ratios in Sealed Bottles for MSR-1 and QH-2 Cultures

Prior to the experiments carried out with the MOCD, the oxygenation conditions were optimized for each strain by varying the “culture medium/air-headspace-bottle” volume ratios. For the MSR-1 strain, 155 mL-serum bottles were prepared with 20, 30, 40, and 50 mL of culture medium. For the QH-2 strain, 155 mL-serum bottles were prepared with 70 mL of culture medium and 260 mL-serum bottles were prepared with 100, 150, 170, and 200 mL of medium. The cultures performed under the optimal conditions thus determined were subsequently used as “culture-references” to evaluate the performance of the MOCD-cultures.

#### Preparation of Dedicated MOCD-Bottles for MSR-1 and QH-2 Cultures

The MOCD-bottles mainly differed from the culture-references described above by the use of (i) recirculation tubes on the bottle-plugs, and (ii) nitrogen (N_2_) instead of air in the bottle-headspaces. The replacement of air by N_2_ in the bottles was carried out just before inoculation by flushing the gaseous phase of the bottles with a sterile N_2_-gas stream (O_2_-free N_2_) for 10 min. Following inoculation, the recirculation tubes of the bottles were mounted around the pump-rotor of the MOCD. To cross-compare the oxygen requirements for MSR-1 and QH-2 strains even though the two volumes of culture media were different, the dilution rate (and consequently the hydraulic residence time) was kept constant at about 0.03 min^-1^ (about 30 min, respectively) by adjusting the pump flow rate depending on the culture medium volume. The experimental conditions for varying the level of oxygenation in MOCD-bottles are detailed below (Table 1). For information, each set of cultures, including the culture-reference and the differently oxygenated MOCD-cultures, were sampled every 10 h (gas and liquid). Furthermore, each culture set was repeated at least three times.

**Table 1:** MOCD-bottles preparation for MSR-1 and QH-2 cultures.

**Strain**	**MSR-1**				**QH-2**				
O_2_-indicator mM h^-1^	1.0	2.0	4.8	9.0	0.02	0.18	0.36	0.72	4.8
Tube-length m	0.5	1.0	2.5	5.0	1.0	0.5	1.0	2.0	2.5
Tube-material^∗^	V	V	V	V	P	V	V	V	V
Volume of culture medium mL	30	30	30	30	170	170	170	170	30
Bottle size mL	155				260				
Pump debit mL min^-1^	1.0	1.0	1.0	1.0	6.0	6.0	6.0	6.0	1.0


*^∗^Symbols “V*,*” and “P” meant Versilic silicon-tube^®^, and PharMed^®^, respectively*.

### Analytical Methods

Cellular growth was determined by measuring the optical density at 600 nm (OD_600_) with an S2100 Diode array UV-Visible spectrophotometer (WPA Biowave, France). The average magnetic orientation of cell suspensions (magnetism) was assayed by the optical method as described in the Schüler’s publication (Schüler et al., 1995). Cells were aligned at different angles relative to the light beam by means of an external magnetic field. The ratio of the resulting maximum and minimum scattering intensities (*C*_mag_) was previously demonstrated to be well correlated with the average number of magnetic particles per cell and could be used for semi-quantitative assessment of magnetite formation (for practical purposes, *C*_mag_ = 0 was assumed for non-magnetic cells).

The gaseous phase of culture-bottles was analyzed by withdrawing 0.3-mL samples from each bottle-headspace with a gas-tight syringe. In the gaseous samples, N_2_, O_2_, and CO_2_ were measured by using a TCD-GC system (Shimadzu 8A, Japan) equipped with a concentric CTR1 column (Alltech, United States). The system was connected to a computer running WINILAB III software (Perichrom, France) to analyze the chromatograms. Operating conditions were as follows: oven temperature 35°C; detector and injector temperature 100°C; current 60 mA; argon carrier gas at 40 mL min^-1^.

Lactate and succinate concentrations were determined by HPLC. One-mL of culture sample was centrifuged for 5 min at 14,500 rpm, then 20 μL was loaded onto an Animex HPX-87H column (Bio-Rad) set at 35°C and eluted at 0.5 mL min^-1^ with H_2_SO_4_ solution (0.75 mM). Product concentrations were determined with a differential refractometer detector (Shimadzu RID 6 A, Japan) connected to a computer running WINILAB III software (Perichrom, France). All analyses were repeated in triplicate. Lactate and succinate concentrations were estimated within an accuracy of ±2% (M/M). Before each sampling in the culture-bottles, either gaseous or liquid, one equi-volume of sterile N_2_ was systematically injected to prevent a change in gaseous pressure inside the culture-bottle.

## Results and Discussion

### Description and Operation of the Micro-Oxygenated Culture Device (MOCD)

**FIGURE 1:**
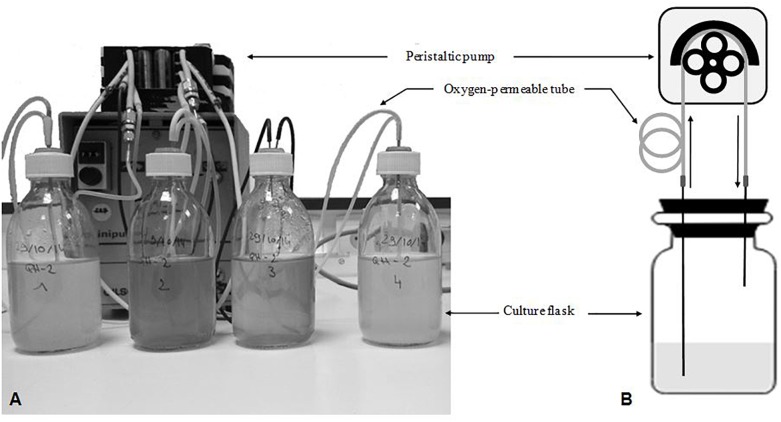
The Micro Oxygenated Culture Device (MOCD). **(A)** Photo and **(B)** Drawing of operating principle. MOCD **(A)** consisted of four cultivation units mounted on a multi-channel peristaltic pump. Each MOCD cultivation unit was equipped with oxygen-permeable-flexible tube arranged around the pump-rotor. The drawing **(B)** shows the interconnection of the main process-elements such as pump rotor, O_2_-permeable flexible tube, and culture-serum bottle.

The MOCD consists of four culture-serum bottles, each connected to an oxygen-permeable-flexible tube looped around the rotor of a peristaltic pump (Figure 1A). The culture-oxygenation is provided by atmospheric oxygen diffusing through the wall of the flexible tube (or recirculation-tube). As shown in Figure 1B, when the pump is running, the culture is sucked from the bottom of the bottle and pumped back to the top of the bottle. For each round, the culture is gradually enriched in oxygen during the transfer along the flexible tube. Back in the bottle, the oxygen content is diluted and consumed by microbial respiration. The amplitude of the oxygen gradient between the entrance and exit of the recirculation-tube is modulated by both the physical parameters (such as pump flow rate, oxygen-permeability of the tube-wall, and geometrical features of the tube) and the respiratory activity of microorganisms. As an option, temperature of the culture could be regulated by immersion of culture-bottles in an open thermostatic bath.

### Model for Oxygen Diffusion Into the Culture Along the Recirculation-Tube

**FIGURE 2:**
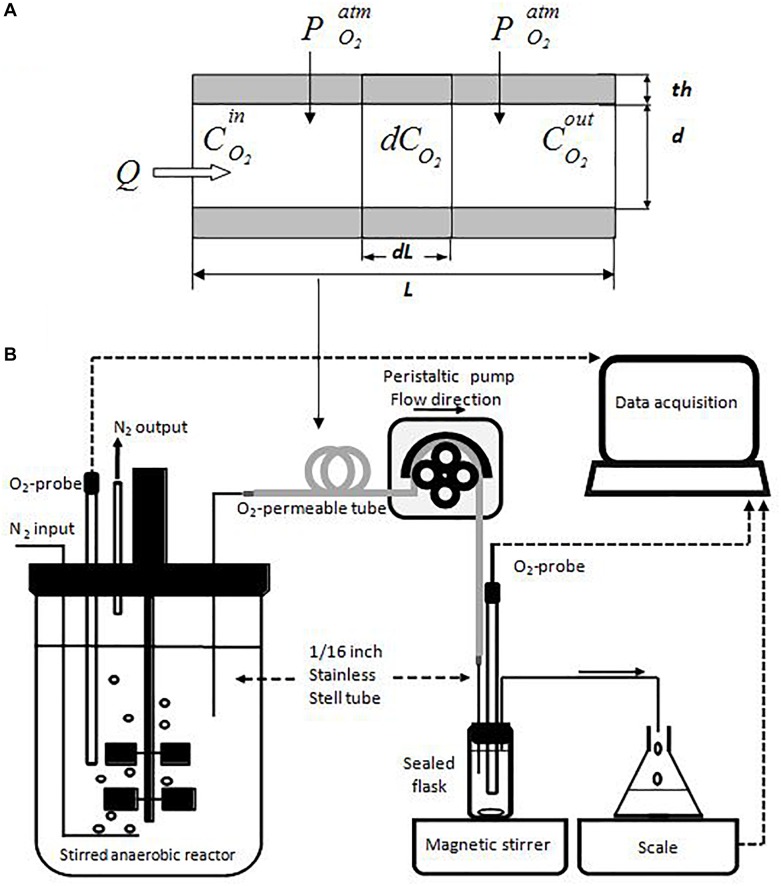
The conceptual model of oxygen diffusion through O_2_-permeable tube **(A)** and the experimental device for model validation **(B)**. In **(A)**, “*Q*” was the water flowing through the O_2_-permeable tube (in gray), “$P_{O_{2}}^{\mathrm{atm}}$” was the oxygen partial pressure at the outside of tube (21%). “$C_{O_{2}}^{\mathrm{in}}$,” “C_O_2__,” and “$C_{O_{2}}^{\mathrm{out}}$” were oxygen concentration changes inside flexible-tube between the entrance and exit. “*th*,” “*d*,” and “*L*” were the wall-thickness, internal diameter and length of tube, respectively. As shown in **(B)**, the flexible tube investigated (in gray) was connected between the reservoir containing anoxic water and the small flask equipped with an O_2_-probe. The dissolved oxygen measured by the O_2_-probe only came from its diffusion through the wall of the flexible tube.

In order to determine the gradual development of O_2_-concentration along the O_2_-permeable tubes of the MOCD, a theoretical mathematical model was built, and a specific experimental setup was designed to validate the model (Figure 2A,B).

The model was mainly based on the Henry’s and first Fick’s laws for gas-diffusion. Assuming that O_2_-concentration was radially homogeneous inside the tube, the O_2_ transfer through the tube-wall was therefore described by equation 1. In this equation, “*P*” was the coefficient of O_2_-permeability only depending on tube-material properties, “*V*” and “*S*” the internal volume and surface of tube, respectively, and “*th*” the thickness of tube-wall. “*C*^s^” was equal to 100% and corresponded to the O_2_-saturation in water under air-atmosphere condition. According to Henry’s law (equation 2), “*Kh*” was the proportionality factor for the dissolved oxygen concentration in water and pO_2_ in the gaseous phase (for the Henry’s coefficients “*Kh*” see Benson and Krause, 1984). In equation 3, “*Q*” was the water flow circulating inside the tube. Equation 4 was achieved by combining equations 1 and 3. Equation 5 was the integral form of equation 4 with “*C*^in^” and “*C^out^*” corresponding to O_2_-concentrations at the entrance and exit of the tube, respectively (Figure 2A).

(1) $$dC\;\times \;dV\;=\;\frac{P}{th\;\times \;Kh}\;\times \;dS\;\times \;\left(C^{s}\;-\;C\right)\;\times \;dt$$

$$dV\;=\;dL\;\times \;\pi \;\times \;{\left(\frac{d}{2}\right)}^{2}$$

dS = dL × π × d

(2) $$Kh\;=\;\frac{C_{O_{2}}}{P_{O_{2}}}$$

(3) $$Q\;=\;\frac{dV}{dt}$$

(4) $$\frac{dC}{dL}\;=\;\frac{P\;\times \;\pi \;\times \;d}{th\;\times \;Kh\;\times \;Q}\;\times \;\left(C^{s}\;-\;C\right)$$

(5) $$ln\left(\frac{C^{s}\;-\;C^{\mathrm{out}}}{C^{s}\;-\;C^{\mathrm{in}}}\right)\;=\;-P\;\times \;\frac{\pi \;\times \;d}{th\;\times \;Kh}\;\times \;\frac{L}{Q}$$

#### Experimental Determination of the Gradual Development of O_2_-Concentration Along Three Kinds of Different O_2_-Permeable Tubes

**FIGURE 3:**
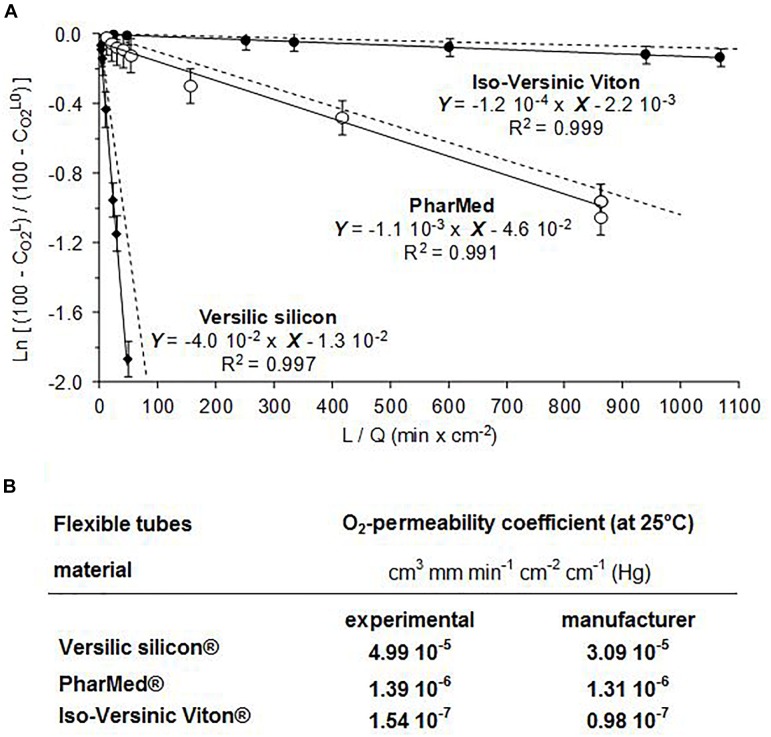
**(A)** Least squares linear regressions with experimental results (-) and theoretical data computed from the mathematical model (---) of O_2_-diffusion through three kinds of tubes. **(B)** O_2_-permeability coefficients of the materials determined experimentally as shown in **(A)** versus given by manufacturers. Symbols (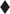, 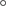, and •) corresponded to experimental measurements (setup Figure 2B) obtained with the tubes made in Versilic-silicon^®^, PharMed^®^, and Iso-Versinic Viton^®^.

The dissolved oxygen measurements reported in Table 2 and plotted in the form of equation 5 (see above) in Figure 3A, were obtained from the experimental setup (Figure 2B) sequentially equipped with the three tubes made in Versilic silicon^®^, PharMed^®^, and Iso-Versinic Viton^®^. These materials have significantly different oxygen permeability features. As shown in Figure 3A, for each tube the “$ln\;\left(\frac{C^{s}\;-\;C^{\mathrm{out}}}{C^{s}\;-\;C^{\mathrm{in}}}\right)$” and the ratio “*L/Q*” exhibited a linear dependence. In addition, the three linear regressions were shown to converge unambiguously toward zero when the ratio “*L/Q*” decreased to zero. This dual outcome fully validated the O_2_-gas transfer model to explain the O_2_-diffusion through the wall of tubes used for the experiments. As indicated by equation 5, given that the slopes of the linear regressions were proportional to the O_2_-permeability coefficient of the tube-materials, the steepest slope corresponded to the highest O_2_-permeable tube-material (Figure 3A). In addition, it was revealed in Figure 3A that the theoretical data (dashed lines), calculated by taking into account geometrical tube-features and O_2_-permeability coefficient values given by the tubing-manufacturers, closely fitted the experimental data. In quantitative terms, this result demonstrated that the methodology combining the O_2_-gas transfer model with the experimental setup (Figure 2B) enabled a correct measurement of O_2_-permeability coefficients for the three tube-materials (Figure 2B). Based on these results, it could be concluded that the methodology described in this section was relevant for estimating the O_2_-permeability of any tube-material under physicochemical conditions close to those used to cultivate microaerobes.

**Table 2:** Dissolved O_2_ measurements for the three tubes made from Iso-Versinic Viton^®^, PharMed^®^, and Versilic-silicone^®^.

O_2_-permeable tube (*d* ×*th*)	*L*	*Q*	*C^in^*	*C^out^*
	*cm*	cm^3^ min^-1^	%	%
Iso-Versinic Viton^®^ (1 mm × 1 mm)	1000	1.06	0.0	10.9
	45	0.95	0.0	0.7
	45	1.84	0.0	0.3
	950	0.89	0.0	12.4
	950	1.58	0.0	7.1
	950	2.84	0.0	4.4
	950	3.78	0.0	3.5
PharMed^®^ (1.6 mm × 1.6 mm)	84	7.17	0.0	1.6
	84	4.08	0.0	5.0
	170	5.75	0.0	7.2
	170	4.23	0.0	8.4
	170	1.09	0.0	25.6
	170	3.16	0.0	11.5
	750	0.87	0.0	65.1
	750	0.87	0.0	61.7
	750	1.80	0.0	38.0
Versilic-silicone^®^ (1 mm × 1 mm)	77	1.61	0.0	84.5
	77	2.73	0.0	68.2
	77	3.50	0.0	61.3
	50	4.99	0.0	34.9
	7	1.83	0.3	13.1
	7	3.05	0.2	8.3
	7	4.28	0.1	6.0


*Size of tubes: “d”, “th,” and “L” meant interior diameter, wall thickness, and length, respectively*. *“Q” was water debit in tubes. “C^*in*^” and “C^*out*^”, expressed in percentage relative to the concentration of oxygen-saturated water (at 25°C and under 1 bar of air), were the oxygen concentrations measured at the entrance and exit of the tube, respectively. PharMed^®^, Versilic-silicone^®^, and Iso-Versinic Viton^®^ were the tube-materials investigated*.

#### Gradients of O_2_-Concentration Along Versilic silicon^®^ and PharMed^®^ Tubes Calculated by Using the Mathematical Model

**FIGURE 4:**
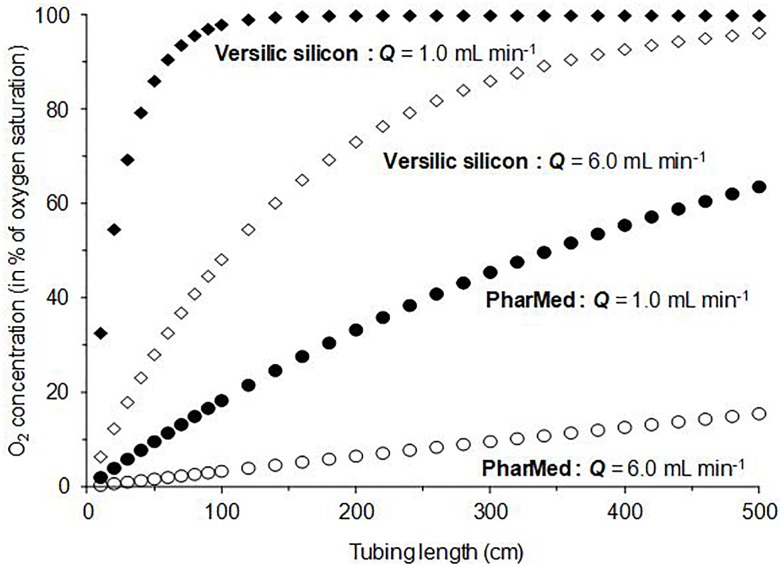
Theoretical gradients of dissolved oxygen along tubes made in Versilic silicon^®^ (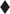, 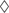), and PharMed^®^ (•, 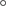). Black and open symbols corresponded, respectively, to 1.0- and 6.0-mL min^-1^ as water flow “*Q*.”

The mathematical model described above (equations 1 and 3) was used to predict the gradients of the O_2_-concentration in the water flowing through the tubes during the operation of the MOCD. As shown in Figure 4, the simulations were only performed with flow rates of 1- and 6-mL min^-1^, lengths of 0–5 m, and restricted to Versilic-silicon^®^ and PharMed^®^. These values as well as the tube-materials were chosen to be as close as possible to the experimental conditions subsequently performed to grow the MSR-1 and QH-2 strains. The curve analysis (Figure 4) clearly showed that the exposure to oxygenation conditions could be largely modulated, in terms of O_2_-concentrations and/or duration, by adjusting the tube-length, pump-flow rate, and/or tube material. Based on these simulations, it has been theoretically demonstrated that the MOCD device enables the user to design and study various ranges of oxygenation conditions.

### MOCD Biological Validation by *Magnetospirillum gryphiswaldense* (Strain MSR-1) and *Magnetospira* sp. (Strain QH-2) Growth

#### Designing an O_2_-Indicator Specifying the Oxygenation Levels of the MOCD-Bottles

To reflect the different oxygenation levels of the MOCD-bottles whatever the selected configuration (tube-length, O_2_-permeability of the recirculation tube, and culture-volume), an O_2_-indicator was specifically designed. Deduced from equation (1), the O_2_-indicator represents the maximal O_2_-flux able to diffuse into the MOCD-bottles per culture volume unit and time unit (mmole of O_2_ L^-1^ h^-1^). As shown in the formula below, it takes into account the O_2_-permeability coefficient of the tube material (as reported in Figure 3B), the tube geometrical features, and the volume of culture medium.

$$O_{2}\;\mathrm{indicator}\;\times \;0.41\;=\;\frac{P\;\times \;S}{th\;\times \;V^{\mathrm{tot}}}\;\times \;16.0$$

In this formula, the unit of the O_2_ permeability coefficient “*P*” was the same as in Figure 3B [cm^3^ mm min^-1^ cm^-2^ cm^-1^ (Hg)]. The units of the internal surface of tube “*S*,” the thickness of tube-walls “*th*,” and the culture volume “*V^tot^*” were in cm^2^, mm, and L, respectively. The value “16.0,” expressed in Hg-cm, corresponded to the pO_2_-difference between outside/inside the recirculation tube, and “0.41” was used as coefficient to express “*O_2_ indicator*” in mmole of O_2_ per liter and per hour (or mM h^-1^). For the O_2_-indicator to be as relevant as possible for all culture conditions, the hydraulic residence time had to be constant. To do this, the flow rate was adjusted depending on the medium volume. For the bottles containing 30 and 170-mL medium, the hydraulic residence time was adjusted to about 28–30 min by tuning the pump-debits to 1.0- and 6.0-mL min^-1^, respectively.

#### Preliminary Experiments: Optimization of MSR-1 and QH-2 Growth in Sealed Bottles

It should be kept in mind that, under our experimental conditions, (i) for MSR-1, lactate was the sole available carbon and energy source in the culture medium, and oxygen was the only available electron acceptor, (ii) for QH-2, succinate and oxygen were the electrons donor and acceptor, respectively.

Prior to experiments carried out with the MOCD, the oxygenation conditions in sealed serum bottles were optimized for MSR-1 and QH-2 growth by varying the volume ratio “culture medium/air-headspace-bottle.” The optimal growth was gained (i) for MSR-1 strain, in the 155-mL serum bottles with 0.24 as volume ratio, i.e., 30-mL culture medium and 125-mL air, and (ii) for QH-2 strain, in the 260-mL serum bottles with 1.9 as volume ratio, i.e., 170-mL culture medium and 90-mL air. The volume ratios found were therefore consistent with the O_2_-sensitivities of the strains, since the highest was favorable for QH-2, the most oxygen sensitive (1.9 for QH-2 versus 0.24 for MSR-1). The cultures performing under optimal conditions were subsequently used to evaluate the performances of the MOCD-cultures.

The bottles dedicated to the MOCD only differed from previous ones by using (i) recirculation tubes and (ii) nitrogen (N_2_) instead of air in the bottle-headspaces (see section “Materials and Methods”). The initial anoxia was chosen to protect these O_2_-sensitive microorganisms against oxygen toxicity. The O_2_-sensitivity of the inoculum is critical before the growth phase, when cells are few and weakly active. With this processing, the MOCD-bottles were gradually oxygenated by O_2_-supply through the recirculation tube.

#### MOCD Efficiency for the MSR-1 Growth

**FIGURE 5:**
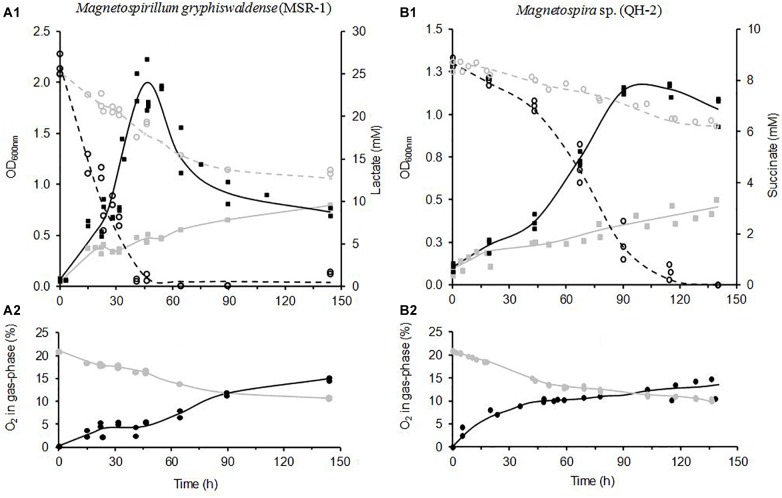
Comparison of *Magnetospirillum gryphiswaldense* (strain MSR-1) **(A)** and *Magnetospira* sp. (strain QH-2) **(B)** cultures performed in sealed bottles used as reference (gray symbols) and in MOCD-bottles (black symbols). MOCD cultures presented here were achieved under optimal O_2_-conditions (2.0 and 0.72 mM h^-1^ for MSR-1 and QH-2, respectively). **(A1,B1)** Represented both growths (solid lines) and energy-substrate consumptions (lactate and succinate, respectively) (dashed lines), and **(A2,B2)** represented the O_2_-changes measured in the gaseous phase of culture-bottles.

**FIGURE 6:**
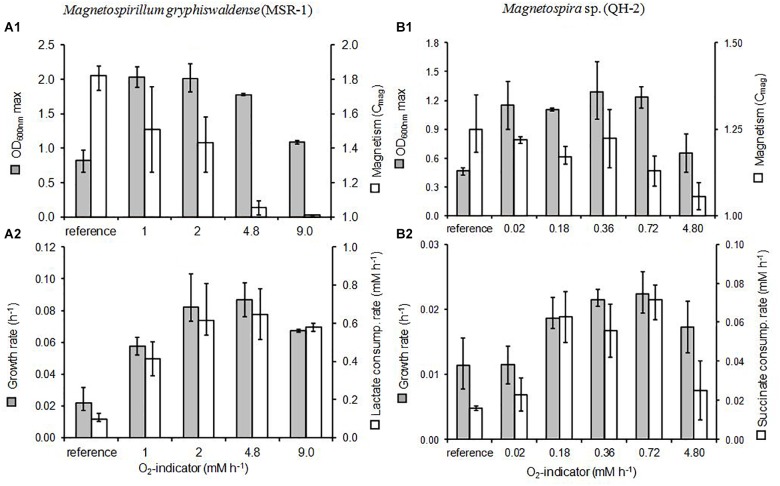
Growth parameters and cellular magnetism in *M*. *gryphiswaldense* (strain MSR-1) **(A)** and *Magnetospira* sp. (strain QH-2) **(B)** grown under different oxygenation conditions. The growth rates, lactate and succinate consumption rates **(A2,B2)** were estimated between initial and maximum OD values measured at 600 nm **(A1,B1)**. Cellular magnetism **(A1,B1)** was estimated by the *C*_mag_ (1.0 indicated no magnetism). “Reference” corresponded to growths performed in sealed bottles with air in the headspace. The “O_2_-indicator” estimated the O_2_-supply provided by the MOCD depending on adjustments of the culture volume, the tube-material, and the geometrical features of tube (see details in sections “Materials and Methods,” and “Results and Discussion”). The sets of cultures including the culture-reference and the MOCD-cultures were repeated three to seven times and the dispersal of results was indicated by error bars.

To investigate the oxygenation ability of the MOCD relative to the MSR-1 growth, different oxygenation conditions were tested by varying the recirculation tube-length (Versilic silicon-tubes^®^) from 0.5 to 5.0 m. The MOCD-bottles equipped with the shortest (0.5 m) and the longest (5.0 m) length supplied, respectively, the lowest and highest oxygenation conditions as indicated by the O_2_-indicator levels (1.0 and 9.0 mM h^-1^, respectively). In Figure 5A1,A2, the parameters were shown for both culture-reference and optimally oxygenated MOCD-culture (O_2_-indicator 2.0 mM h^-1^). Parameters for all cultures were reported in Figure 6A1,A2 to compare the effect of oxygenation levels on growth and magnetism of the strain MSR-1.

Cross comparisons of culture parameters (Figure 6A1,A2) such as maximal cell concentration (OD_max_), growth rate, and lactate consumption rate proved unambiguously that the best oxygenation condition for MSR-1 growth was reached with the MOCD O_2_-indicator level 2.0 mM h^-1^ (tube-length 1.0 m) (MOCD-culture “opt”). Under this condition, growth and lactate consumption rates of the MOCD-culture “opt” were six and fourfold higher than those of the reference culture, respectively (Figure 6A2). Figure 5A1 showed that MOCD-culture “opt” consumed the entire medium lactate (25 mM) in about 40 h while the reference culture only consumed 12-mM of lactate after 150 h. The low cell concentration obtained for the reference culture (0.8 units of OD_600_ versus 2.0 for MOCD-culture “opt”) resulted from the limiting effect of oxygen on the growth and lactate consumption rates (Figure 6A1).

The cross-comparison of all MOCD MSR-1 cultures (Figure 6A2) revealed that shortening the Versilic silicon^®^ tube-length from 1.0 to 0.5 m (O_2_-ind. from 2.0 to 1.0 mM h^-1^, respectively) resulted in the limitation of MSR-1 growth by oxygen. For the MOCD–culture O_2_-ind. 1.0, both growth and lactate consumption rates were significantly slowed down by about one third compared to the MOCD-culture “opt.” However, the maximum cell concentrations reached were found to be similar in both cases (Figure 6A1). Otherwise, it was striking to note that some oxygen was accumulated within all headspaces of MOCD-bottles during the MSR-1 growth whatever the oxygenation conditions were including the O_2_-limited one represented by the MOCD-culture O_2_-ind. 1.0. The O_2_-accumulation kinetics for all MOCD-cultures of MSR-1 were found close to the one plotted in Figure 5A2. Despite strong cell growth, oxygen was found to accumulate in all MOCD-bottle headspaces. The O_2_-contents reached 4–5% when cell concentrations reached their maxima as shown in Figure 5A2. Based on these results it can be hypothesized that, when the MOCD was working, the O_2_-enriched culture medium flowing in the recirculation-tube and dropping in the bottle released a fraction of dissolved oxygen within the bottle headspaces. This phenomenon should be all the stronger as the atmosphere, initially O_2_-free, remained low in oxygen during cell growth. In addition, the operating conditions (low O2-content in bottle-atmosphere and unstirred liquid phase), limiting oxygen transfer from the gaseous to the liquid phase, promoted the oxygen accumulation in bottle headspaces. Therefore, the degassed oxygen accumulated in bottle-atmosphere should be considered as trapped and unavailable for microorganisms. As a supplementary argument, the weak oxygen transfer was verified in the reference culture. Indeed, although the atmosphere was richer in O_2_ (initially 21%) the fact that the liquid was not stirred was the major physical factor that restrained the O_2_-transfer (Figure 5A2).

At the other end of the oxygenation spectrum, t O_2_-toxicity in MSR-1 was clearly exhibited with the highest oxygenated MOCD-culture (O_2_-ind. 9.0) obtained with 5.0 m of Versilic silicon-tube^®^. Compared to the MOCD-culture “opt,” the toxic effect of O_2_ was demonstrated by the significant decrease in both growth and lactate consumption rates (Figure 6A2), but also by the drop in maximum cell concentration (1.1 versus 2.0 units OD_600__nm_) (Figure 6A1). Under this O_2_-toxic condition (MOCD-culture O_2_-ind. 9.0), MSR-1 needed 140–150 h to consume the 25 mM of lactate initially present, versus only 40 h for the MOCD-culture “opt.”

##### Performances of the MOCD versus the oxystatbioreactors for MSR-1 growth

To further evaluate the performance of the MOCD for MSR-1 culture, our results were compared to those of Heyen and Schüler and used as the reference (Heyen and Schüler, 2003). To study the effects of oxygen on MSR-1 cell growth and magnetite production, these authors have specially designed a sophisticated oxystat-bioreactor to rigorously control oxygen partial pressure (pO_2_) within a range from 0.25 to 212 mbar (or 0.1–100% of the O_2_-saturation in water at 28°C under air-atmosphere). Comparing their results to ours was all the more relevant and easy as the culture medium formulations used to cultivate the MSR-1 strain were very similar. Heyen and Schüler demonstrated that the MSR-1 growth was (i) optimal for any pO_2_ level ranging from 0.25 to 151 mbar (0.1–70% of O_2_ satured), and (ii) partially inhibited at a pO_2_ level close to saturation (beyond 212 mbar or 90% of O_2_-satured). Under optimal oxygenation condition, they estimated the growth rate and the cell yield on lactate at 0.13 h^-1^ and 14.5 g M^-1^ (Heyen and Schüler, 2003).

Although much less sophisticated than the oxystat-bioreactor designed by Heyen and Schüler, the MOCD gave a good performance regarding the growth rate (0.08–0.09 h^-1^ with MOCD-culture “opt” versus 0.13 h^-1^ with the Heyen and Schüler’s oxystat-bioreactor) (Figure 6A2). Unexpectedly, the optimum cell yield on lactate reached with the MOCD (24.8 g M^-1^ for cultures “MOCD O_2_-ind. 1.0” and MOCD “opt”) was significantly higher than with the oxystat-bioreactor (14.5 g M^-1^) (Heyen and Schüler, 2003). It is noteworthy that other authors, using a bioreactor optimized for O_2_ supply but with a slightly different culture medium [enriched in yeast extract (0.1 g L^-1^)], got a very similar estimation for MSR-1 cell yield on lactate (14.8 g M^-1^) (Heyen and Schüler, 2003). The reasons for the significantly higher cell yield obtained with the MOCD versus the oxystat-bioreactors for the MSR-1 strain remains unclear. It might be noted that the high heterogeneity in time and space of the oxygen concentrations distributed in the culture medium is a major difference between MOCD cultures and well-homogenized cultures performed in bioreactors (due to combined effects of both mechanical stirring and gas diffusion). During the MOCD process O_2_-gradients were *de facto* developed into the culture medium along the recirculation-tube and between the surface and bottom of culture bottles. It could therefore be hypothesized that the heterogeneity of oxygen concentrations produced by the MOCD in the culture medium could be favorable to the MSR-1 strain growth.

#### MOCD Efficiency for the Magnetite Production in MSR-1

The response of MSR-1 cells to magnetism (*C*_mag_) was inversely proportional to the level of oxygenation of cultures (as shown by O_2_-indicator) (Figure 6A1). The response to magnetism was maximal when cell growth was limited by oxygen. In contrast, no response was measured when the oxygenation conditions were toxic to growth (O_2_-ind. 9.0)(Figure 6A1,A2). The magnetism estimations (*C*_mag_) achieved by spectrophotometer were consistent with the observations of magnetosomes under Transmission Electron Microscope (Supplementary Figures S1A,B). The magnetite crystals, forming the magnetosome chains inside MSR-1 cells, were more mature and numerous in cells grown in poorly oxygenated MOCD-bottles (O_2_-ind. 1.0) than in those grown under higher oxygenated conditions (O_2_-ind. 9.0) (Supplementary Figures S1A,B). These results were found fully consistent with the conclusions of work published so far in this issue (Blakemore et al., 1985; Schüler and Baeuerlein, 1998; Yang et al., 2001; Heyen and Schüler, 2003; Sun et al., 2008). Therefore, the O_2_-tuning of the MOCD enabled the discrimination and determination of MSR-1 specific oxygenation conditions for cell magnetism and cell growth.

#### MOCD Efficiency for Growth and Magnetite Production in the Microaerobic Strain QH-2

As shown for the MSR-1 MOCD-cultures and despite the higher O_2_-sensitivity of the QH-2 strain, it was demonstrated that the MOCD allowed the determination of three specific conditions of oxygenation for QH-2 growth, i.e., optimal, toxic and limiting. These specific oxygenation conditions were determined by changing the tube material (PharMed^®^ or Versilic silicon-tube^®^), tube-length (from 0.5 to 2.5 m), and the volume of culture medium (170 or 30 mL), as detailed in Table 1 of the Section “Materials and Methods.” Thus, based on maximum cell concentration (OD_600_ max), growth rate, and succinate consumption rate, the oxygenation condition was found to be limiting, optimal (MOCD-culture “opt”), and toxic when the O_2_-indicator level of the MOCD was down to 0.18 mM h^-1^, ranged from 0.18 to 0.72 mM h^-1^, and up to 0.72 mM h^-1^, respectively (Figure 6B1,B2). Cross-comparison of the MOCD-culture “opt” with the culture-reference revealed unambiguously that the MOCD significantly increased the growth rate by about twofold (0.36 versus 0.11 h^-1^) and the succinate consumption rate by about threefold (0.72 versus 0.02 mM h^-1^) (Figure 6B2). The MOCD performances for QH-2 growth could also be appreciated by comparing the MOCD-culture “opt” to the culture-reference as shown in Figure 5B1,B2. Figure 5B1 showed that the MOCD-culture “opt” consumed all the succinate initially present for 140 h while in the reference culture 6 mM succinate still remained after more than 140 h of incubation. Consequently, the maximum cell concentration of the culture-reference only reached 0.46 units of OD_600_ versus 1.3 units for the MOCD-culture “opt” (Figure 5B1, 6B1).

Despite the very high O_2_ sensitivity of QH-2, the MOCD delivered accurate enough oxygenation conditions for magnetite production and cell growth. As the *C*_mag_ assessments showed (Figure 6B1), the cell’s magnetic response was optimal for O_2_-indicators between 0.02- and 0.36-mM h^-1^ and gradually decreased for higher indicator levels. As previously observed in MSR-1, QH-2 cell’s magnetism was dramatically inhibited when oxygen became toxic for growth (O_2_ ind. 4.8) (Figure 6B1). Transmission Electron Microscopy of the cells revealed that only the less oxygenated conditions (O_2_-indicator from 0.02 to 0.36 mM.h^-1^) led to biomineralization of numerous and typically shaped magnetosomes (Supplementary Figures S2A,B).

#### Discussion About the MSR-1 and QH-2 O2-Sensitivity Estimations Determined With the MOCD

On the basis of growth carried out in oxystat bioreactors, the O_2_-toxicity thresholds for MSR-1 and QH-2 growth were estimated at 210 μM for dissolved oxygen (Heyen and Schüler, 2003; Sun et al., 2008), and 40 μM (our unpublished results), respectively. Beyond these threshold-levels, the O_2_-toxicity resulted in both a decrease in growth and the inhibition of magnetosome formation. Even though the MOCD (O2 indicator) and the oxystat bioreactor technologies were different, they led to similar conclusions regarding the O_2_-sensitivities of the MSR-1 and QH-2 strains. Based on the MOCD cultures, QH-2 strain consistently exhibited a higher O_2_ sensitivity than MSR-1 (optimally growth from 0.18 to 0.72 mM h^-1^ for QH-2 versus 2.0 to 4.8 mM h^-1^ for MSR-1) (Figure 6A2,B2). Furthermore, it was good to note that the MOCD-O_2_-indicator values determined for the O_2_-toxicity thresholds for MSR-1 and QH-2 growth were close to the results delivered by the oxystat bioreactors in terms of proportionality. The O_2_-toxicity thresholds determined by the MOCD for MSR-1 and QH-2, revealed that QH-2 was sixfold lower than MSR-1 (0.72 vs. 4.8 mM h^-1^, respectively). By comparison, the results delivered with the oxystat bioreactor indicated an O_2_-toxicity threshold for QH-2 growth fivefold lower than for MSR-1 (O_2_-concentrations up to 40 and 210 μM, respectively). Unlike the oxystat-bioreactors the MOCD did not measure oxygen tensions. However, the MOCD-O_2_-indicator was accurate enough to be used to characterize and classify microorganisms or physiological functions (such as cell magnetism) depending on the O_2_-sensitivity.

## Conclusion

The results obtained from this work demonstrated that, the MOCD significantly improved the growth of the two microaerobic magnetotactic bacteria strains (QH-2 and MSR-1) i.e., the growth rates increased by two and fourfold, respectively, when compared to the traditional cultivation methods using simple sealed bottles. In addition, when comparing the results reported in the literature for MSR-1 growth, the performance of the MOCD was found to be close to or even better than the oxystat bioreactors when considering cellular yield. Moreover, the O_2_-conditions that could be obtained with the different MOCD-configurations were suitable and accurate enough to distinguish optimal O_2_-conditions for growth and magnetite production in both QH-2 and MSR-1 strains despite their different oxygen requirements. Lastly, the O_2_-indicator, a specifically designed parameter to distinguish and discriminate all oxygenation conditions whatever the MOCD configuration was, proved to be reliable enough to estimate the specific O_2_-sensitivity of MSR-1 and QH-2 strains.

Based on the performance established during this work and on the wide variety of oxygenation conditions that it could be adjusted to, the MOCD should be considered as a novel and promising tool for growing and studying all microaerobes whatever their oxygen requirements. Its low cost and capacity to integrate serial growing-bottles make it a powerful tool for studying microaerobes. Therefore, using the MOCD gives scientists the opportunity to carry out various work including ecophysiology, ecology, biodiversity, and description of the microflora within all the microoxic environments.

## Author Contributions

MF performed most of the experiments, analyzed the data, and revised the manuscript. SD supervised experimental part of the project and participated in results analysis. CB performed experiments particularly for MSR-1 growing, analyzed the data, and revised the manuscript. L-FW was involved in the design of the experimental study using QH-2 as strain and revised the manuscript. YC-B acted as coordinator of the project, designed this study, analyzed experimental results, drafted and revised the manuscript.

## Conflict of Interest Statement

The authors declare that the research was conducted in the absence of any commercial or financial relationships that could be construed as a potential conflict of interest.
