# Supplementary figures and images for: A Novel Highly Efficient Device for Growing Micro-Aerophilic Microorganisms
Source: Front Microbiol. 2019 Mar 19;10:534. doi: 10.3389/fmicb.2019.00534 (PMC6434946; doi:10.3389/fmicb.2019.00534)

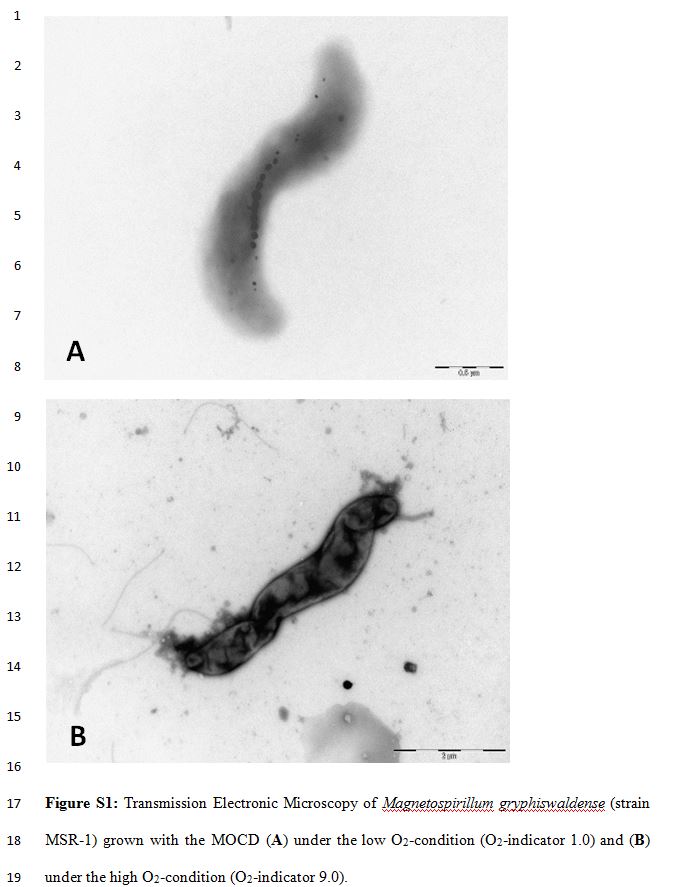

Supplement: Supplementary file 1 [file Image_1.JPEG]

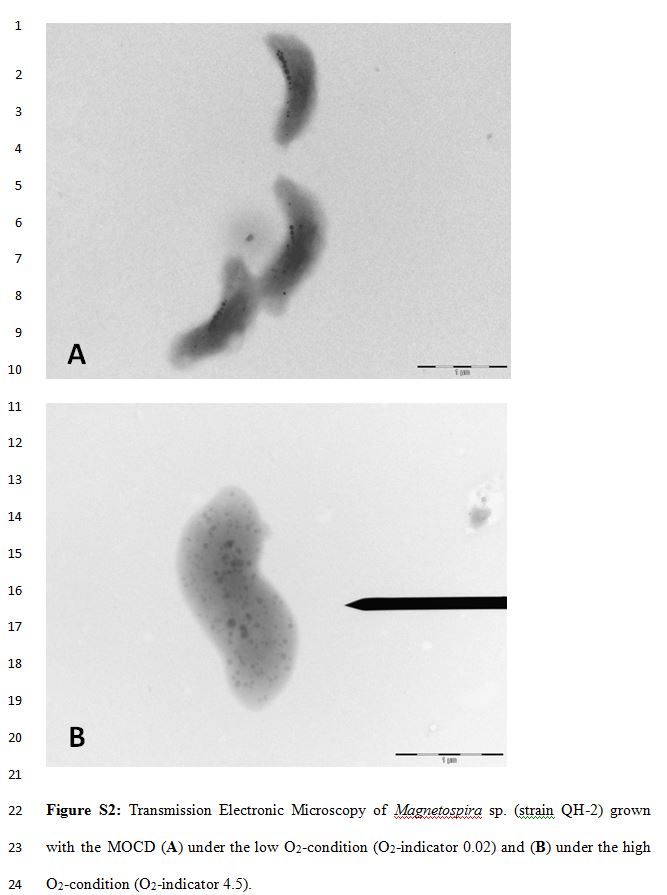

Supplement: Supplementary file 2 [file Image_2.JPEG]
